# Supplementary material for: Genome-Wide Association Study for Agronomic Traits in Gamma-Ray-Derived Mutant Kenaf (Hibiscus cannabinus L.)
Source: Plants (Basel). 2024 Jan 16;13(2):249. doi: 10.3390/plants13020249 (PMC10819814; doi:10.3390/plants13020249)
Supplement: Supplementary file 1 [file plants-13-00249-s001.zip › Figure S1.pdf]

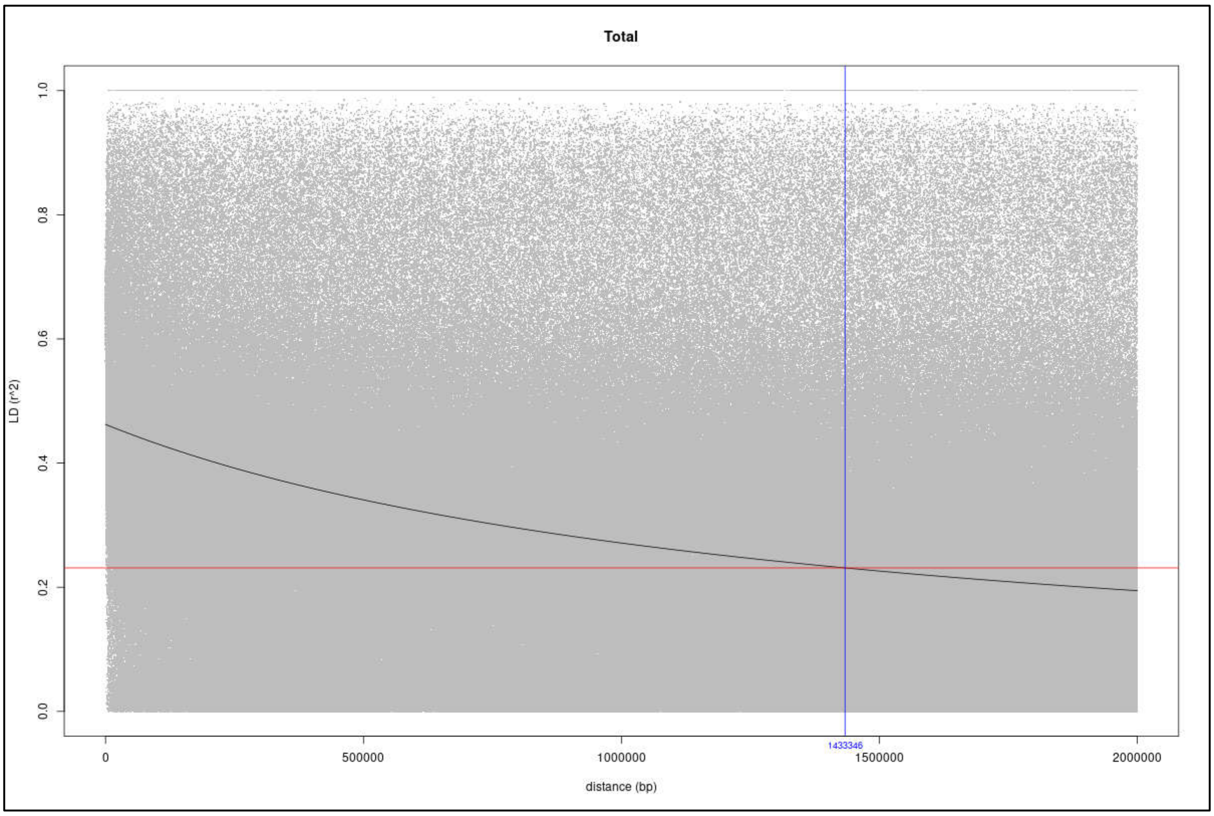

Supplementary figure 1. Distribution of the marker density and decay of the linkage disequilibrium (LD) over distance presented as an accumulative distribution.
