# Supplementary material for: Genome-Wide Association Study for Agronomic Traits in Gamma-Ray-Derived Mutant Kenaf (Hibiscus cannabinus L.)
Source: Plants (Basel). 2024 Jan 16;13(2):249. doi: 10.3390/plants13020249 (PMC10819814; doi:10.3390/plants13020249)
Supplement: Supplementary file 1 [file plants-13-00249-s001.zip › Table S1.pdf]

Table S1. Country of origin for the 96 kenaf assessed in this study.

Sample	Country	Origin variety	Treatment	Generation
C9	Russia			
C10	India			
C11	Iran			
C12	Italy			
C13	Russia			
C14	Italy			
C15	China			
C16	China			
C17	China			
C18	China			
C19	India			
2012_WFM_1_2		C14	Natural variation	M7
2012_WFM_1_5		C14	Natural variation	M7
2012_WFM_2_1		C14	Natural variation	M7
2012_WFM_2_3		C14	Natural variation	M7
ACC4111	Bangladesh			
ACC4649	Bangladesh			
ACC5014	Bangladesh			
ACC5072	Bangladesh			
ACC5113	Bangladesh			
A16		Auxu	Gamma ray	M7
ACC4443	Bangladesh			
ACC4985	Bangladesh			
ACC5047	Bangladesh			
ACC5077	Bangladesh			
A18		Auxu	Gamma ray	M7
A13		Auxu	Gamma ray	M7
A1_2		Auxu	Gamma ray	M7
A11		Auxu	Gamma ray	M7
gimjejinju300_2		Jinju	Gamma ray	M9
gimjejinju300		Jinju	Gamma ray	M9
wonbaek_jangbaek31		Baekma	Gamma ray	M7
A13_SP		Auxu	Gamma ray	M7
A22		Auxu	Gamma ray	M7
A19_dae		Auxu	Gamma ray	M7
Everglades41	USA			
gimjejinju300_1		Jinju	Gamma ray	M9
RS_1		C14	Gamma ray	M7
jangbaek_316		Baekma	Gamma ray	M7
jangbaek_72		Baekma	Gamma ray	M7
jangbaek_whiteflower_F14		JangdaeXBaekma	Cross	F8
T2_L		Jinju	Gamma ray	M5
W2		Jinju	Gamma ray	M5
W8		Jinju	Gamma ray	M5
Auxu22		Auxu	Gamma ray	M7
C_11_P	Iran		Gamma ray	
V2		Jinju	Gamma ray	M5
Baekma		C14	Gamma ray	
jangdae		Jinju	Gamma ray	
bora		Hongma300	Gamma ray	M5
Hongma_300	China			
Hongma_743	China			
Auxu	China			
Auxu_SP2_bio		Auxu	Gamma ray	M7
2012_WFM_1_3		C14	Natural variation	M7
2012_WFM_1_4		C14	Natural variation	M7

Auxu_SP		Auxu	Gamma ray	M7
Jinju	Korea			
Hongma_300_300Gy		Hongma300	Gamma ray	M5
2012_WFM_1_6		C14	Natural variation	M7
A12		Auxu	Gamma ray	M7
2012_WFM_2_2		C14	Natural variation	M7
ACC3748	Bangladesh			
ACC4139	Bangladesh			
ACC4751	Bangladesh			
ACC5017	Bangladesh			
ACC4153	Bangladesh			
A1		Auxu	Gamma ray	M7
A8_2		Auxu	Gamma ray	M7
gimjejinju300_3		Jinju	Gamma ray	M9
A14		Auxu	Gamma ray	M7
A20		Auxu	Gamma ray	M7
C14_RS1		C14	Gamma ray	M7
jangXbaek_whiteflower_F14_21		JangdaeXBaekma	Cross	F8
jeokXbaek_66		JeokbongXBaekma	Cross	F8
T3		Jinju	Gamma ray	M5
Z_1		Jinju	Gamma ray	M5
C14_RS2		C14	Gamma ray	M7
A22_3		Auxu	Gamma ray	M7
jangXbaek_66		JangdaeXBaekma	Cross	F8
jangjeok_S25		Jeokbong	Gamma ray	M7
V3_whiteflower		Jinju	Gamma ray	M5
jangXbaek_21a		JangdaeXBaekma	Cross	F8
T4		Jinju	Gamma ray	M5
jinjuXbaekma		JinjuXBaekma	Cross	F8
wandae		Jinju	Gamma ray	M5
jangjeok_S26		Jeokbong	Gamma ray	M7
V7		Jinju	Gamma ray	M5
Z_4		Jinju	Gamma ray	M5
jeokbong		C14	Gamma ray	
jangbaek_7		Baekma	Gamma ray	M7
jangbaek_2a_3		Baekma	Gamma ray	M7
jangjeok_S7		Baekma	Gamma ray	M7
jangbaek_18		Baekma	Gamma ray	M7
132_dae_3		Baekma	Gamma ray	M5
jangbaek_2a_baeksaek_2		Baekma	Gamma ray	M5
